# Association of Autoimmune Hepatitis and Celiac Disease: Role of Gluten-Free Diet in Reversing Liver Dysfunction

**DOI:** 10.1177/2324709617705679

**Published:** 2017-04-19

**Authors:** Umair Iqbal, Ahmad Chaudhary, Muhammad Arsalan Karim, Muhammad Arsalan Siddiqui, Hafsa Anwar, Nancy Merrell

**Affiliations:** 1 Bassett Medical Center, Cooperstown, NY, USA; 2 Dow University of Health and Sciences, Karachi, Pakistan; 3 Henry Ford Hospital, Detroit, MI, USA

**Keywords:** celiac disease, gluten-sensitive enteropathy, autoimmune hepatitis, gluten-free diet

## Abstract

Autoimmune hepatitis (AIH) is a chronic inflammation of liver with unclear etiology. It is frequently associated other autoimmune diseases, and its association with celiac disease (CD) is well established. In this article, we describe the case of a 50-year-old male with long-standing AIH taking azathioprine for 10 years, evaluated for flares in transaminases. Despite adding high-dose corticosteroids, his transaminases and bilirubin remained high. Serology for CD was ordered, which revealed elevated tissue transglutaminase antibody IgG and endomysial IgA, which was further confirmed by endoscopic biopsy. Strict gluten-free diet was advised and now for over 2 years he is in remission with azathioprine and budesonide. This emphasizes the role of gluten-free diet in reversing liver dysfunction in patients with AIH, and clinicians should consider screening for CD in patients with AIH with persistent elevation of liver enzymes despite immunosuppressant treatment.

## Introduction

Celiac disease (CD), also called gluten-sensitive enteropathy, is an autoimmune disease triggered by the ingestion of the gliadin fraction of gluten and/or other cereal prolamins in genetically predisposed patients.^1^ Although the small bowel is the primary target manifesting as diarrhea, flatulence, and weight loss due to malabsorption, extensive evidence shows that CD is a systemic disorder affecting the liver, thyroid, pancreas, connective tissue, bone, heart, skin, and nervous system.^1^ The most common liver manifestation in CD is isolated hypertransaminasemia,^2^ while autoimmune-related manifestations like autoimmune hepatitis (AIH), primary biliary cirrhosis, and so on, are also common.^3^ AIH is an idiopathic chronic inflammation of the liver, frequently associated with CD.^1,4-6^ In this article, we report a case of AIH that presented to us with recurrent flares of hypertransaminasemia. Extensive hepatic workup along with workup for CD showed celiac-related AIH, and immunosuppression along with strict gluten-free diet (GFD) helped achieve remission.

## Case

**Figure 1.**
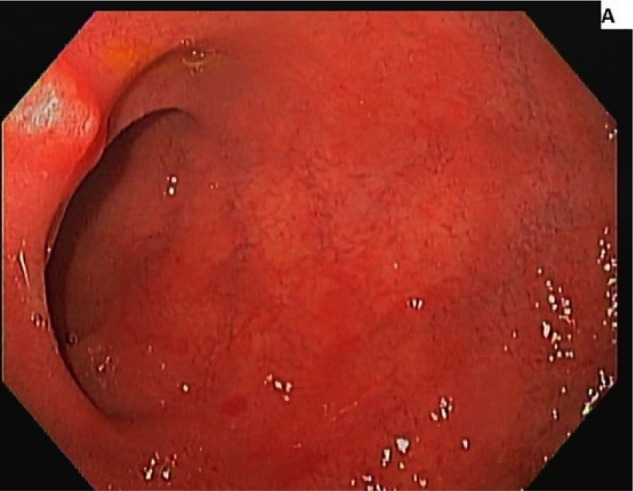
Distal duodenum showing complete absence of villi. Endoscopic biopsy of duodenum.

**Figure 2.**
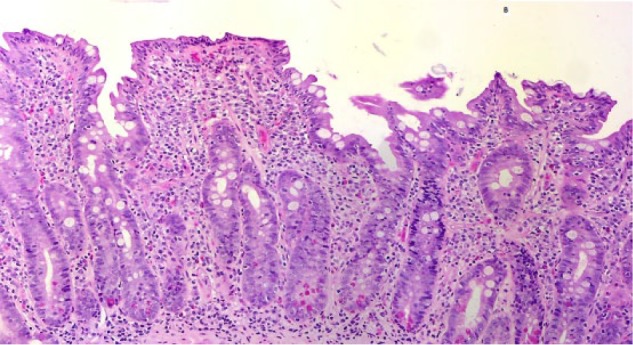
Histopathology showing villous flattening with some residual wide and blunted villi. Presence of intraepithelial lymphocytes is consistent with diagnosis of celiac disease.

A 50-year-old male with a history of hypertension and long-standing AIH taking azathioprine for 10 years was evaluated for flares in transaminases, aspartate transaminase 123 U/L, alanine transaminase 115 U/L, bilirubin 2.6 mg/dL, international normalized ratio 1.3, and thrombocytopenia. There was no relevant family, alcohol, or drug history, and he had never received a blood transfusion. He denies any use of over-the-counter drugs or herbal supplements. Serum markers for hepatitis A virus, hepatitis B virus, and hepatitis C virus were negative. Serum antinuclear antibodies were found to be positive but anti-smooth muscle antibodies and anti-mitochondrial antibodies were negative, as were antibodies against cytomegalovirus or Epstein-Barr virus. He had normal iron, thyroid-stimulating hormone, α-1 antitrypsin, and ceruloplasmin. Abdominal ultrasound showed heterogeneous and nodular liver. Liver biopsy showed grade 3 portal fibrosis concerning for cirrhosis, which could also be the likely cause of his thrombocytopenia. Budesonide was added to his drug regimen. Despite high-dose steroids, his transaminases remained high and bilirubin continued to rise. He did not show any signs and symptoms of decompensated liver disease. Serology for CD was done and revealed elevated tissue transglutaminase antibodies (tissue transglutaminase immunoglobulin A = 26.2 U/L; tissue transglutaminase immunoglobulin G = 11.9 U/L) and positive endomysial immunoglobulin A antibody. Endoscopic biopsy of duodenum showed flattening of villi with intraepithelial lymphocytes, which confirmed the diagnosis of CD (Figures 1 and 2). Strict GFD was advised resulting in normalization of transaminases and bilirubin in a repeat laboratory workup in 2 months. Now for over 2 years he is in remission on azathioprine, budesonide, and strict GFD (see Table 1).

**Table 1.** Liver Chemistry Results Before and After the Initiation of Gluten-Free Diet (GFD).

Liver Function Test	Reference Range	Pre-GFD	3 Months Post-GFD	6 Months Post-GFD
AST	Latest reference range: 17-59 U/L	123	63	52
ALT	Latest reference range: 21-72 U/L	115	71	57
Total bilirubin	Latest reference range: 0-1.0 mg/dL	2.6	2.6	2.0

Abbreviations: GFD, gluten-free diet; AST, aspartate transaminase; ALT, alanine transaminase.

## Discussion

Celiac disease is associated with many autoimmune diseases like type 1 diabetes mellitus, autoimmune thyroiditis, psoriasis, AIH, inflammatory bowel disease, vitiligo, and so on.^5^ Conversely, the prevalence of CD in AIH is significantly higher than that found in the general population, with 2 studies reporting the prevalence as 6.4% and 4%.^4,7^ AIH and CD share a common immunological basis. Approximately 95% of CD patients express *HLA-DQ2*, and the remaining patients have *HLA-DQ8*.^8^ *HLA-DR3* expressed in AIH has a strong linkage with *HLA-DQ2* and may account for the association between these diseases.^9^ This linkage probably explains damage to hepatocytes as well as the intestinal mucosa by the immunologic mechanism generated by tTG acting both as a self-antigen or generating neo-antigen.^4,10^

Studies demonstrate that early serological screening testing for CD is strongly recommended in all patients with AIH.^4,8,10^ Commonly used serological tests used for detecting CD are anti-gliadin antibodies, anti-endomysial antibodies(EmA), and anti-tTG antibodies. Anti-gliadin antibody testing is no longer used routinely because of its low specificity and sensitivity. The IgA anti-tTG is an optimal screening test with its 96% sensitivity, and IgA-EmA is diagnostic (specificity nearly 100%). Intestinal biopsy findings remain the gold standard and are used to confirm positive antibody test results. In children aged less than 5 years, EmA and tTG antibody testing are less reliable and require additional study.^11^

GFD is an effective treatment for most patients with CD and extraintestinal manifestations.^9,10,12-14^ The role of GFD particularly in patients with AIH is still not fully explained. Kaukinen et al^10^ demonstrated in their study that 3 out of 4 patients with severe liver disease and concomitant CD were found to be no longer meeting the criteria for liver transplantation after maintained on a GFD, with reversal of hepatic dysfunction noticed in all 4 patients. A possible explanation of the benefit from GFD is that it heals the gastrointestinal mucosa and decreases any further triggers of autoimmunity.^15^ In another study, GFD along with steroids resulted in higher remission rates in pediatric patients with AIH coexisting with CD, compared with AIH without CD.^16^

Rubio-Tapia^17^ from their study delineate that a portion of adults that developed a complete mucosal recovery could have a lower risk of all-cause mortality than patients with persistent mucosal damage. However, they did not find complete mucosal recovery in a significant number of adults with CD, despite considerable improvement in clinical response after treatment with a GFD. This could be explained either due to poor adherence to GFD or gluten from unidentifiable sources, or even a longer duration of study is required in adults for the effect to be measured. On the other hand, children were found to have achieved mucosal recovery as much as 95% after 2 years of treatment with a GFD.^17^

In summary, growing evidence suggests considerable benefits of GFD in decreasing the risk of subsequent autoimmune disease, malignancy, osteoporosis,^5^ as well as mortality,^18^ but further studies are required to shed light on its implication in achieving remission in AIH.

## Conclusion

Clinician should consider screening of CD in patients with AIH with persistent elevation of liver enzymes despite immunosuppressant and corticosteroid treatment. Early application of GFD can prevent further liver damage and can help achieve remission in these patients.
